# Treatment of effluents from Food Services Establishment (FSEs) by Physico-chemical processes: a case study for Trinidad & Tobago

**DOI:** 10.1186/s13036-023-00344-w

**Published:** 2024-01-05

**Authors:** Cudjoe Shamika, Banerjee Goutam, Cooper Vincent

**Affiliations:** 1 Department of Civil and Environmental Engineering, The University of The West Indies, St. Augustine, Trinidad & Tobago; 2 Department of Civil Engineering, Indian Institute of Technology (IIT), Varanasi, 221005 India

**Keywords:** Characterization, Fats, Oils and Grease (FOG), Food Services Establishments (FSEs), Coagulation-flocculation, Poly-aluminium Chloride (PAC), Poly-electrolytes

## Abstract

**Background:**

Effluents from Food Services Establishments (FSEs) contain primarily Fats, Oil and Grease (FOG) which severely impact on sewers and the environment when released in high concentrations. In Trinidad & Tobago, it is estimated that approximately 231,304 kg/day of unaccounted for FOG bearing wastewaters from FSEs, are released into the environment with no viable treatment in the country. This research explored the optimization of physico-chemical processes for the treatment of FOGs for subsequent release into sewers.

**Results:**

Bench-scale studies analysed the characteristics of FSE’s effluents from three popular sources, conducted the treatment of these effluents using Jar Tests, and subsequently confirm results via a pilot plant study. Characterization showed the mean concentration of the parameters examined to be; FOG (511 mg/l ± 116 mg/l), Suspended Solids (446 mg/l ± 146 mg/l), Chemical Oxygen Demand (2229 mg/l ± 963 mg/l) and pH (6 ± 0.3). Jar Tests were conducted using Poly-aluminium Chloride (PACl) as coagulant, anionic and cationic polyelectrolytes as flocculant aids with suitable pH adjustments of samples to determine the isoelectric point for the coagulant. Effluent results showed FOG removal levels of 99.9% and final effluent concentration of 0.17 mg/l. This was attained using PACl concentration of 250 mg/l, a 0.1% low cationic polyelectrolyte (CP 1154) at 4 mg/l with the pH of sample adjusted to 8. The pilot plant achieved a 97.4% removal of FOG (residual of 16.8 mg/l) using the same coagulant dosing, and pH value, but increasing the strength of the flocculant aid to 0.1% medium cationic (CP1156) at 5 mg/l.

**Conclusion:**

Experimentation showed high concentrations of emulsified FOG can be efficiently removed to levels below the permissible requirements (20 mg/l) for entry into sewer systems in Trinidad and Tobago using coagulation, flocculation and sedimentation techniques. Pilot scale study also revealed that a higher strength and/or dose of the cationic polyelectrolyte and increased times in primary and final tanks were required to attain the desired results as in the bench level study, where equipment limitations in the flocculation tank were faced. This is in alignment with theory where factors critical for agglomeration is equipment type and density charge. It is, concluded that the optimum combination of chemicals and the respective dosages attained at the bench level study should prove effective should the right equipment be made available.

## Introduction

Fats, oil and grease, collectively known as FOG can severely impact the sewerage system and by extension the environment, if not treated and disposed of properly. FOG constitutes substances of petroleum origin, known as brown oil and those from animal and vegetable origin, known as yellow grease. FOG (yellow and brown), when discharged into the environment in excessive amounts, do not degrade easily resulting in sewer chokes which lead to sewage overflows into the environment. FOG in liquid form appear harmless, however, when it cools it congeals and hardens and attaches to the inner lining of drainage pipes and sewers. Sewer pipes are generally made from hydrophobic and oleophilic materials to which oily substances are naturally attracted; Trinidad and Tobago WASA [1]. As such, once oil and grease cools and congeals it attaches to the inner lining of sewer pipes, which results in a harden formation inside of the pipe, which subsequently takes away from the carrying capacity of the pipe resulting in sewage overflows with time. Sewage overflows pose health problems as sewage carries pathogenic organisms including viruses and also cause damage to properties; Boston Water and Sewer Commission [2]. Moreover, when laden with oil, it causes fire hazards on spillage to environment. In Trinidad, the FSEs are rapidly growing generating more oil and grease to be disposed of. However, the absence of legitimate disposal standards has led to illegal dumping and inappropriate disposal practices.

Subsequent to these chokes additional problems that are encountered include:

- Biological processes used to treat domestic wastewater would be inhibited, resulting in significant expenditure and increase in operations and maintenance costs, to restore them to their effective operational levels. This would include the shutting down of treatment plants and lift stations for servicing and the replacement and repair of equipment damages;
- Costs are incurred by the government and unwarranted inconveniences to the public when rectifications of the problems caused by FOG are needed to be done on the collection system. This is the case as public sewer chokes are very costly and require mobilization of significant amount of resources;
- Operational costs to repair and service wastewater treatment plant where processes have been affected due to excessive inflow of FOG into the wastewater treatment plant;
- Resultant increase in operation and maintenance costs along with loss of revenue due to loss of production in Food Services Establishments (FSEs);
- Reduction of revenue from loss of customers due to the obnoxious odours produced internally to building drains of Commercial Businesses;
- Because of chokes in the sewer line, anaerobic condition can be a result which can lead to releases of hydrogen sulphide which are a fire hazards;
- Explosion of rodent and vermin population thereby increasing risks of the transmittance of communicable diseases.

In Trinidad and Tobago, the Food Services Establishments (FSEs) are rapidly growing and as such, there are larger quantities of oil and grease to be disposed of daily. However, the absence of legitimate disposal has led to illegal dumping and inappropriate disposal practices as discussed over personal communication with local authorities. Control by the relevant Authorities to mitigate these issues is also unnoticeable as enforcement of policies are not rigid. There are many incidences of excessive amounts of FOG entering the public sewer system, causing sewage overflows into the environment in Trinidad and Tobago. It has been reported by the local authorities that most of these grease problems have been caused by absence of adequate pre- treatment systems in some FSEs, either grease traps/interceptors being non-existent, or these are poorly designed and/or ill-maintained. Data reported for Trinidad show that there is approximately 59% of FOG wastewater unaccounted for in Trinidad on a daily basis via records from the Wastewater Department up to 2011 from the Trinidad Wastewater Utility. For an approximate specific gravity of 997,000 mg/l for FOG in water, the equivalent weight for unaccounted FOG is approximately 231,304 kg daily. This figure definitely shows the magnitude of the FOG problem.

The novelty of this project is based on the characterization and determination of treatment for emulsified wastewater from FSEs in Trinidad, in the absence of a proven viable treatment system in the country. In order to ascertain the best footing to start treatment, a literature survey was conducted. Literature survey showed that this problem is also international and various attempts have been made to find an effective and efficient disposal system over the years. These vary from gravity separation methods, membrane and thermal processes to biological and physico-chemical treatment methods to reduce FOG concentrations. Research for papers were pulled from several databases and streamlined from 1997 to 2014, with most of the studies being conducted 2000 onwards. Research for solutions to the said problem was also done regionally and locally via the West Indian Database at the Univeristy of the West Indies St. Augustine and available infromation from private and government agencies. However, this level of research showed no substantiated proven methods/solutions. Subsequently, further investigations were done via field work at FOG generating industries in Trinidad and Tobago. There it was revealed that FOG treatment explored in Trinidad ranged amongst microbial, dissolved aeration flotation (coagulation and flocculation included), polishing ponds and soil beds. Whislt treatment results were not disclosed, operational and maintenance costs were indicated as high and in some cases an alternative method was being explored. Further, locally there was a treatment plant installed in Trinidad that was approved and operated for FOG from FSEs using bacteria/ aeration processes. Liaison with the water and wastewater utility of Trinidad and Tobago, namely, Water and Sewerage Authority (WASA), revealed that these processes were not effective (the effluent which was released into the public sewer system was often high in FOG concentration resulting in oveflowing sewage on the collection system and high amounts of FOG at one of the major wastewater treatment plants) and the plant was eventually decomissioned. It should be noted efforts were used to meet compliance, by adding a gravity type interceptor at the end of the plant processing units and before final discharge into the sewer system. However, both biological and physical methods still proved futile.

Table 1 gives a summary of some of the more successful methods used to treat different types of FOGs and reflects the effectiveness of each method.

**Table 1:** Showing Different Methods of Treatment Used to Treat Various Fats Oils and Grease (yellow)

Reference	Type FOG	Type Treatment	Achievement
Pintor et al. [3] and He [4]	Emulsified FOG from VOR	Gravitational Methods	80% Removal in 30 min
Vinci [5]	Effluent from Wastewater Plant at Miami Dade Water and Sewer Dept.	Pilot Plant using Filter Beds with High Level Disinfection, Reverse Osmosis, MF and Ultra Violet Disinfection with Advanced Oxidation	1-11 mg/l went to 1–3 mg/l (72%)
Shon et al. [6]	Animal fat, Sunflow and Olive Oil	Bacterial – lipase Bacterium D_2_D_3_ with aid of yeast extract and peptane	62–94% for detention times greater than 24 h.
Xu and Zhu [7]	FOG from Refectory	Electrocoagulation manipulating electrode distances	Over 95% FOG and 75% COD for 30 min detention at 10 mm distance
Bayramoglu et al. [8]	Poultry Slaughter House	Electrocoagulation manipulating material of electrodes	Iron electrode removed 98% FOG but Aluminium achieved less in FOG but higher in 95% COD
Qin et al. [9]	FOG from Restaurant	Combined electro-coagulation-electro-flotation (EC-EF) with quadratic programming system	Zero Trade Effluent

In concluding the literature survey, it is seen the problem to be resolved has been considered by scholars over the last decade. The scope of the problem is wide i.e. FOG, even when specific to vegetable and animal waste, has several types such as from FSEs, vegetable refinery and slaughter houses. Also, for each type there are several aqueous layers where some layers have been studied. Various methods have been explored to treat different forms of animal and vegetable grease wastewater. Some articles speak specifically to emulsified and others settables, while most speak in general of FOG. The most consistent and effective results were seen from the physico-chemical treatment processes which are dominated by electro coagulation and flocculation. For those specific to wastewater from FSEs biological methods were explored locally and were found to be unsatisfactory. In international studies, optimum results were attained via a sophisticated experimental type set up [9] that used a pilot plant with the aid of a centralized quadratic program which predetermined the optimum dosage requirement and therefore resulted in zero trade effluent. It should be noted that none of these address directly the treatment of the emulsified FOG from FSEs.

Based on these problems and noting there is no successful conventional treatment system reported for the disposal of this type of wastewater in Trinidad coupled with the studies stemming from the Literature Survey, this paper embarks into treatment of the emulsified layer (notably the toughest layer) of FOG-laden FSE effluents by physico-chemical means, viz. pre-sedimentation followed by coagulation-flocculation-sedimentation.

## Experimental Programme

### Sampling

Three FSEs with the most popular food processing operations were identified for sampling. They constituted predominantly frying (chicken and chips), predominantly baking (pizzeria) and predominantly cooking of various dishes using combination of all cooking processes. Grab samples were manually collected from the grease traps of the three FSEs, placed in a portable deep freezer and carried to the laboratory within thirty minutes. It shall be noted that the collected samples were also composited in proportions which would be representative of what a hauler would have for disposal. Sampling for characterization of FOG for each aforementioned FSE was done once per week, with one proportionally mixed sample (homogeneous mix of three (3) individual samples from each of the three (3) FSEs) were taken fortnightly. In general, sampling for analysis of treatment at the Bench Level Studies and Pilot Scale Studies were done weekly over a two (2) month period each, with one proportionally mixed sample to be analysed for each test run.

### Analysis

The first phase of analysis (Bench Level Studies) of the grab samples from individual FSEs effluents, as well as the second phase of analysis of composite samples were analyzed for pH, Total Suspended Solids (TSS), Biochemical Oxygen Demand (BOD), Chemical Oxygen Demand (COD), and FOG as per Standard Methods [10]. Each extraction of emulsified water to be tested was placed in a 2L beaker and was continuously stirred via submergence of a magnet in the beaker to ensure homogeneity in sample while conducting tests. Once characterization was completed, the Jar test was performed using different dosages of Poly-aluminium Chloride (PACL), variation of pH adjustments and polyelectrolytes. Prior to Jar Test, the sample was allowed to stand to allow separation of the FOG layers before extracting the emulsified layer for treatment. Following the Jar Test (treatment process), the sample was again allowed to stand before testing of the effluent for aforementioned parameters. Details are discussed under sub heading titled “Bench Level Studies – coagulation and flocculation.”

At the second phase (Pilot Scale Study) a homogeneously mixed volume of the sample was extracted and placed in beaker using aforementioned test methods for characterization. The remaining sample which was examined, in alignment with the bench level procedures. The initial step of separation was attained by allowing the sample to have a quiescent period in the first tank (pre-sedimentation), following which the emulsified layer was extracted via a transfer pipe with gate valve to the second and third tanks, to initiate the physicochemical treatment. The final tank was used for settling of floc after treatment for a quiescent period, before the final effluent was withdrawn and tested. Details are discussed under sub heading titled “Pilot Scale Studies – coagulation and flocculation.” Treatment commenced using the chemical findings from the bench level studies, where further mathematical analysis and subsequent changes in physical and chemical parameters were determined, in order to reproduce efficient results at the pilot scale.

### Bench Level Studies - Coagulation and Flocculation

Jar tests were carried out fortnightly to determine the optimum coagulation-flocculation treatment process using various strengths and dosages of poly-aluminium chloride (PACl) and various proprietary polyelectrolytes (supplied by General Electric). The steps for coagulation-flocculation - settling were as follows:

- A 5% Stock of PACl was made up and placed in the volumetric flask.
- 0.1% stock of the polyelectrolyte used was made up (based on manufacturer’s instructions) and placed in a glass jar.
- The sample collected was poured into a 2-L graduated cylinder and left to settle for 30 min. The subnatant emulsified water was withdrawn and placed in each of the six 1-L beakers.
- Each beaker was placed on the magnetic stirrer with the magnet immersed in the liquid.
- A pipette was used to apply drops of the sodium hydroxide until the desired pH of the sample was attained. This was done whilst the pH probe was immersed in the contents thereby monitoring the pH value.
- The beakers along with the contents therein were replaced on the platform of the six place flocculator. The paddles were then gently immersed in the liquid.
- The respective dosages of PACl were then quickly removed from the volumetric flask through 25 ml pipette and placed in the respective beakers.
- Flash mixing (coagulation) continued for 2 min @ 100 rpm.
- The requisite polyelectrolyte dosages were then quickly pipetted from the glass jar and placed into the respective 6 beakers and the slow mixing (flocculation) was done for 20 min @ 20 rpm.
- Following which the six place flocculator was turned off and the contents left to settle for 30 min.
- The top layer in each beaker was thrown off and the subnatant was used for analysis of FOG, COD, TSS, pH, and aluminium.
- All parameters tested were done in accordance with Standard Methods [10].
- The jar tests were repeated until the optimal dosages of PACl as well as polyelectrolyte combination was determined.

### Pilot Scale Studies – Coagulation and Flocculation

- Prior to treatment the sample was tested for FOG, TSS, COD, pH (to verify characterization).
- The sample collected was gently poured into the first tank of the pilot plant and left to settle (to differentiate between layers) for varying times between 30 and 120 min.
- The Two (2) Cole Palmer Stirrers Propeller Type (Model Numbers 500007-30 and 50002-40), were set up on either side of the length of the coagulation tank, symmetrically positioned to allow homogenous mixing. They were both immersed to a depth of 10% of tank width from the bottom of the tank and at an angle of 10% from the vertical to reduce vortexing.
- The gate valve on the overflow pipe from the first chamber (pre-sedimentation) to the second chamber (coagulation tank) was opened (small opening) to allow the emulsified layer to be transferred to the coagulation tank with minimal disturbance. As such, this regime was considered non-turbulent mixing, despite Reynold’s number not being confirmed in the pipe.
- The stirrers were then put on to mix the water while the pH of the sample was adjusted until a value of 8 was attained. This was done via a pipette used to apply drops of the Sodium Hydroxide and pH indicator strips.
- When the desired pH value was attained 250 mg/l of PACl (pre-determined optimum dosage at bench level) was then quickly removed from a glass jar (with previously mixed coagulant) via the 0.025 × 10^− 3^ m^3^ pipette and placed in the coagulation tank.
- Coagulation then commenced for two (2) minutes at the required rpm. Note determination of the rpm depended on volume of sample, mixing velocities, tip speed and power of stirrers.
- After the two (2) minutes the mechanical stirrers were slowed down to 0 rpm and the contents in the coagulation tank were allowed to flow via gravity to the flocculation tank via a small opening of the gate valve on the transfer pipe. It should be noted the valve was opened to control the flow to minimise disturbances of the flocs formed while throttling to the contents already in the flocculation tank. As such, this regime was considered non-turbulent mixing, despite Reynold’s number not being confirmed in the pipe.
- The two mechanical stirrers were then placed in the same position for the flocculation tank. The polyelectrolyte dosage (previously mixed) were then quickly pipetted from the glass jar into the flocculation tank based on respective dosages.
- Flocculation quickly commenced for twenty (20) minutes at the required rpm. Note determination of the rpm depended on volume of sample, mixing velocities, tip speed and power of stirrers.
- Following which the Two (2) mechanical stirrers were turned off and the contents allowed to transfer to the final tank (settling tank) via small opening of the ball valve on the transfer pipe, so as to minimise disturbances through the pipe and in the receiving tank. The sample was allowed to settle for various times (between 60 and 360 min) following which the supernatant was drawn off to be tested.
- Respective tests were then conducted on the effluent (FOG, COD, TSS, pH, Aluminium). It shall be noted that analysis of aluminium in sludge was done via formula.
- This procedure was carried out until verification of the Bench level studies were attained or equally desired results.

## Results and Discussion

### Characterization of effluents from FSEs

Four pertinent wastewater quality parameters were analysed, viz. combined fats, oil and grease (FOG); total suspended solids (TSS); pH; and chemical oxygen demand (COD). It should be noted that a composite sample (C) was also analysed to represent the realistic situation of what any grease hauler would bring to a wastewater treatment plant for treatment. This sample “C” is the mixture of FSE effluent samples “K” (baking-based), “M” (frying-based) and “W” (mixed-based).

Table 2 summarizes the characteristics of FOG-bearing effluent (emulsified layer) from three FSEs (via composite sample “C”) based on sampling and analyses discussed in the forgoing section.

**Table 2:**
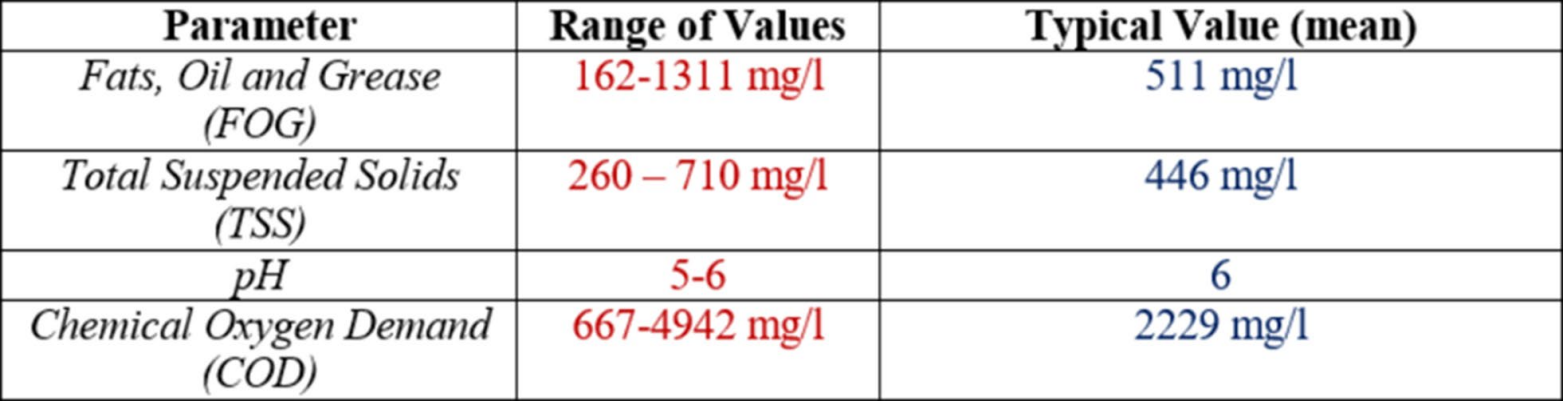
Characteristics of FSE Effluent

It may be noted that the parameter with the highest concentration is COD (average of 2229 mg/l), followed by FOG (average, 511 mg/l) and TSS (average, 446 mg/l). The above results reveal that the combination of samples would be ideal and realistic to be envisaged for its physico – chemical treatment by coagulation-flocculation-sedimentation.

### Bench-scale study

The physico-chemical treatment selected in this study is conventional coagulation, flocculation and settling via Jar Tests. Poly-aluminium Chloride was used to perform coagulation. The effects of additions of poly-electrolytes (supplied by General Electric) were also examined. Poly-aluminium Chloride ([Al(OH)_a_Cl_b_]_n_, where (a + b) = 3, with a > 1.05) (Water New Zealand 2013) [11] is a class of soluble aluminium products where the aluminium chloride has been partially reacted with a base. It contains some of the highly cationic oligomers of aluminium, which makes it an excellent choice for coagulation. Polyelectrolytes are used for floc build up as they bridge between particles and charged aluminium flocs (Eckenfelder Jr. 1966, 280) [12]. They are also absorbable meaning small dosages are effective to encourage floc build up. Colloids of oil and grease are hydrophobic which often retard flocculation and frequently require special treatment to achieve effective coagulation (Eckenfelder Jr. 1966, 280) [12]. Additionally, the vast majority of colloids are negatively charged (with a range of the zeta potential between − 12 to -40 mv). Consequently, coagulation is induced by the addition of high valence cations. Optimum coagulation occurs when the zeta potential is zero (isoelectric point – which occurs at a specific pH for the respective chemical), with effective coagulation at +/- (-5) mv (Eckenfelder Jr. 1966, 283).

**Fig. 1:**
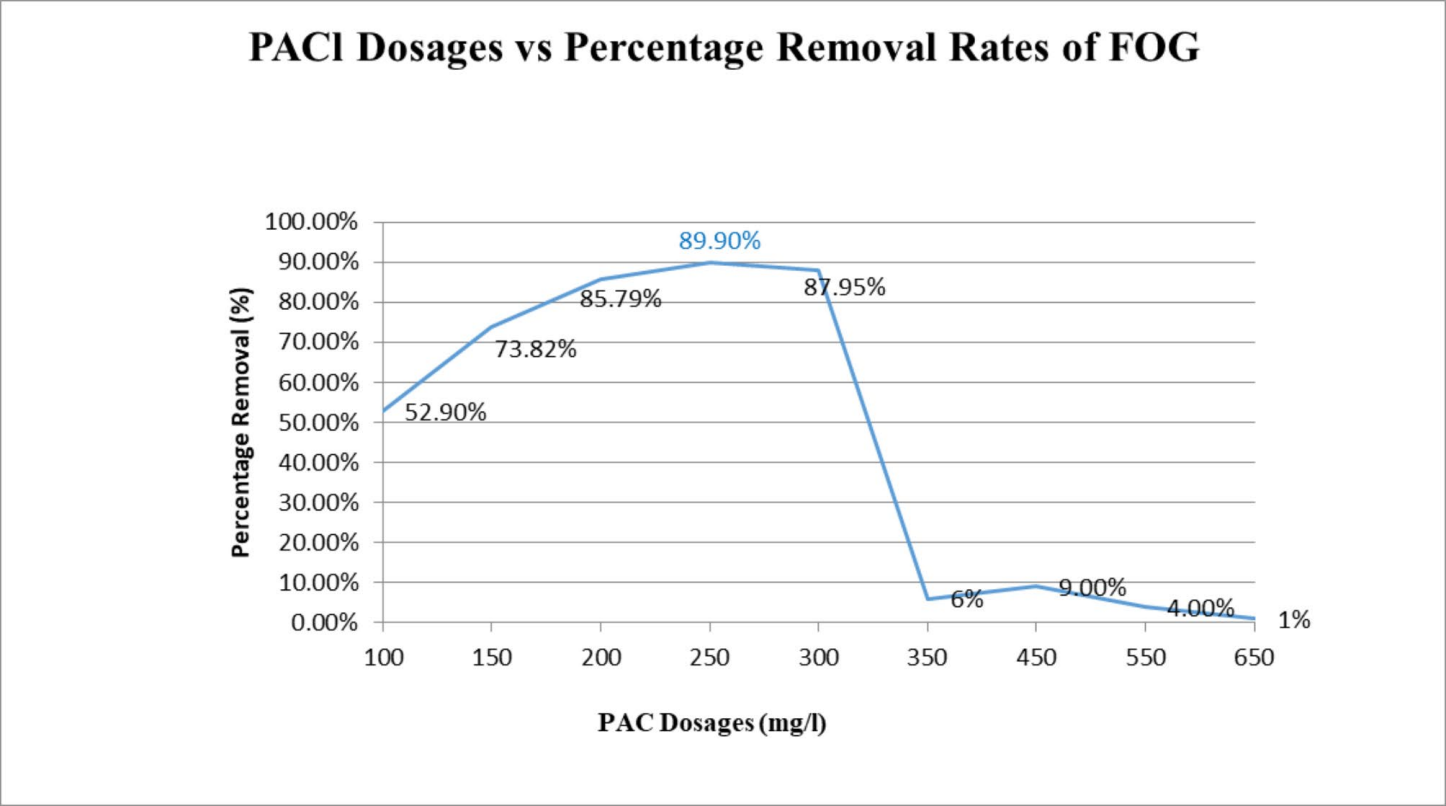
PACl Dosages vs. Percentage Removal of FOG

Figure 1 shows removal rates of FOG at varying dosages (10 0 mg/l – 650 mg/l) of a 5% Stock Solution of PACl subsequent to coagulation and flocculation of the emulsified sample. Better removal rates were seen for dosages ranging between 150 mg/l to 300 mg/l, with a drastic drop in removal for dosages higher than 300 mg/l, which can be an indication that the Zeta Potential closest to zero and subsequent optimal destabilization had occur in a lesser dosage. 250 mg/l dosage shows the optimum removal (89.90%), indicative of the dose which makes the emulsion most unstable. A blank was prepared during experimentation but analysed last, thereby allowing a greater standing time. As such the removal rate for the blank was 80.52% which is indicative of a larger percent of grease being floatables. This suggests that a longer standing time (at least eighty two minutes, which the blank would have stood for before testing) was required before chemical treatment. pH remained on the higher end of the characterization range i.e. 6, which is expected as PAC is reacted on the base end of the pH scale even though carrying hydrogen ions (H+) which contributes to acidity. The removals of TSS ranged between 11 and 65% and that of COD in the range of 52 − 91%.

**Fig. 2:**
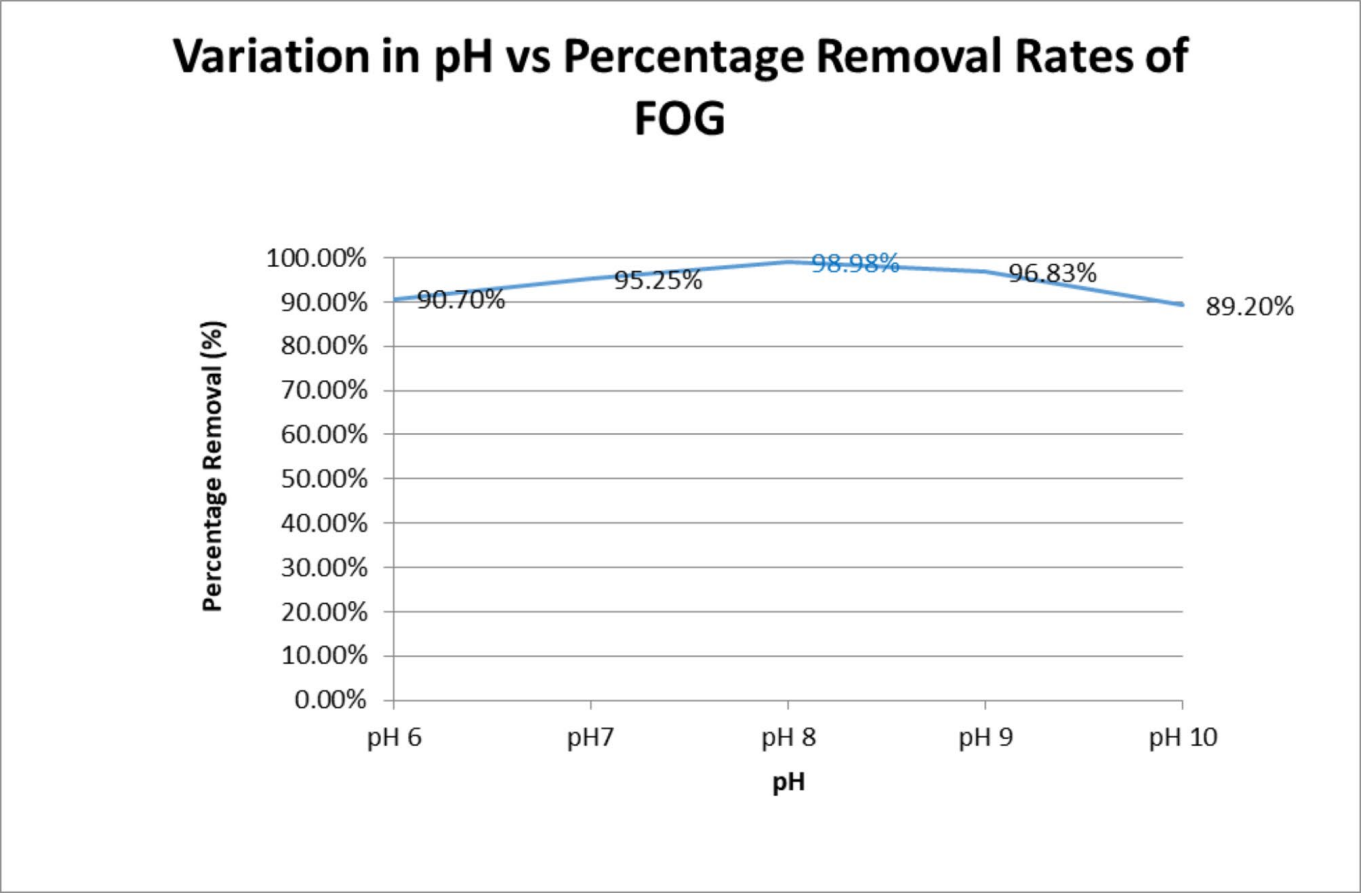
Removal of FOG vs. pH at 250 mg/l of 5% Stock PACl

Figure 2 shows that oil and grease removal rates not only increased with altered pH, but removal rates generally remained above 90% over a pH range of 6–10. Optimum removal of 98.98% was achieved for a pH of 8. TSS removal rates (93–99%) were also in alignment with FOG rates which may be attributed to the increase in destabilisation of colloids. pH range varied between 6 and 8 which is more towards the base end, as expected with the addition of Sodium Hydroxide (NaOH). On the other hand, the upper range of removal COD dropped to 61%, which is expected with the addition of a chemical (NaOH).

**Fig. 3:**
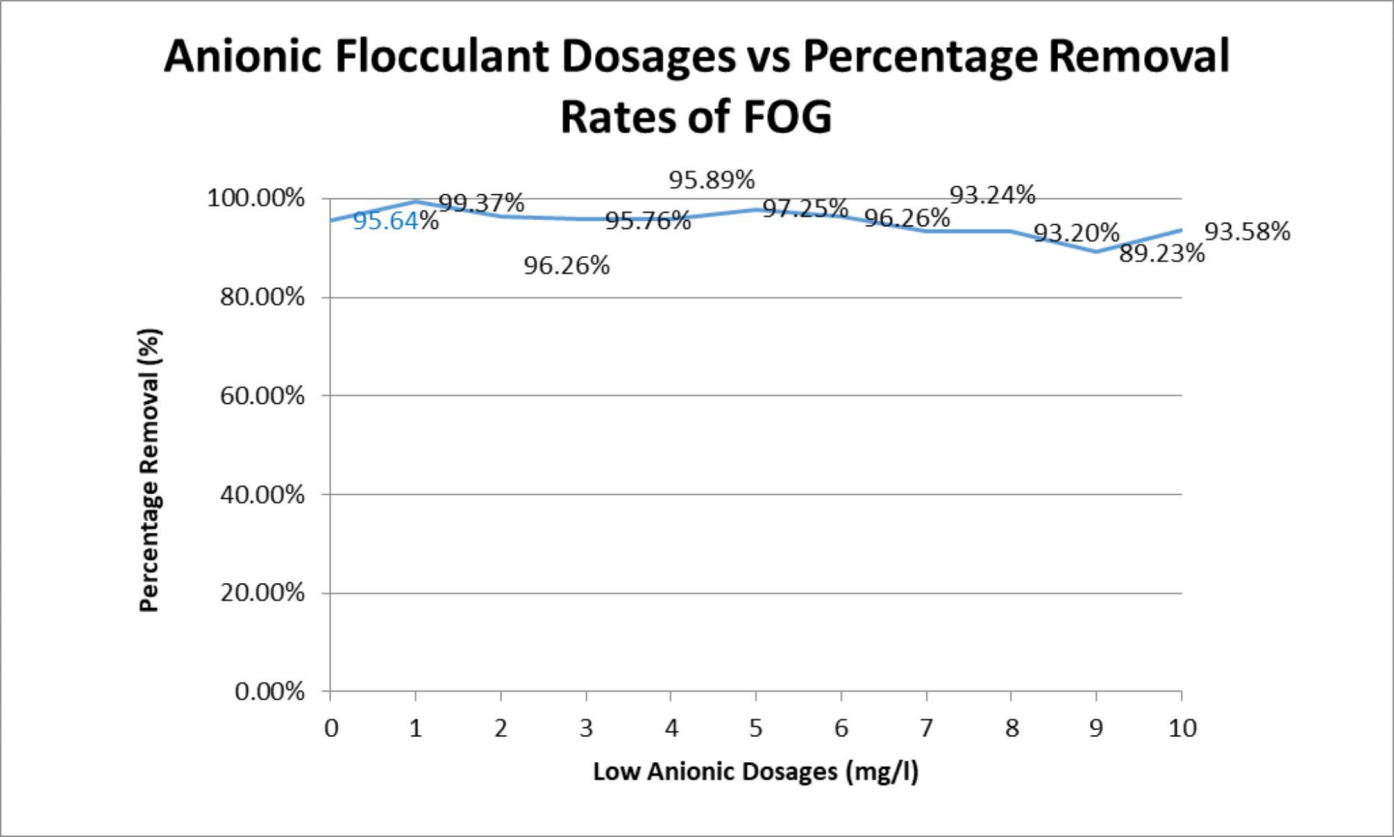
Removal of FOG at various dosages of low anionic polyelectrolyte along with 250 mg/L

Figure 3 illustrates the effects of the optimum PAC dosage (250 mg/L) and pH adjustment of samples to 8, with addition of low anionic polyelectrolyte dosages to assess its efficacy in the treatment. Figure 3 shows there was no significant change in FOG removals with the addition of the low anionic polyelectrolyte, rather, with increased dosages past 1 mg/l, there was a decreased trend in the removal of oil and grease. This is an indication that the probable net charge left after coagulation is negative. TSS removal remained in the 90%, with the exception of the polyelectrolyte dosage of 6 mg/l, which gave a removal of 41%. COD removals generally dropped in a range of 40 − 60% with two high removals of 76% and 85% for 0 mg/l and 7 mg/l of polyelectrolyte added respectively. pH went unaffected by the addition of the polyelectrolyte.

### Of PACl with pH of sample adjusted to 8

**Fig. 4:**
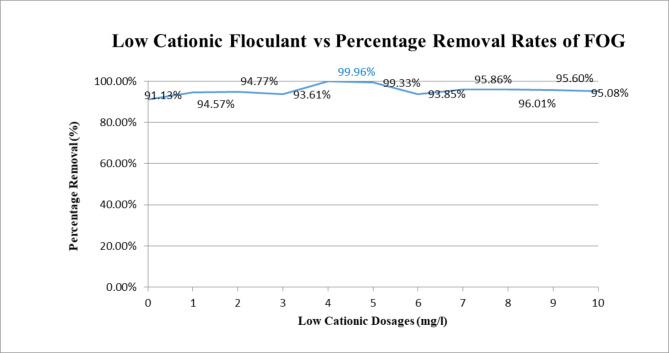
Removal of FOG at various dosages of low cationic polyelectrolyte along with 250 mg/L of

Based on the insignificant change by the addition of the anionic polyelectrolyte, a low cationic polyelectrolyte was tried. Figure 4 shows the results.

### PAC with pH of sample adjusted to 8

It is seen that optimum performance regarding the removal of emulsified oil and grease was achieved with a poly-aluminium chloride (PACl) dosage of 250 mg/l for a 5% Stock with the influent sample adjusted to a pH of 8 (seen to be isoelectric point for PACl) and with a low cationic polyelectrolyte dosage of 4 mg/l. This resulted in a removal rate of 99.9% with an FOG residual of 0.17 mg/l. TSS removals remained in a high range (90 percentile range) as anticipated. However, COD removals significantly dropped because of the polymeric organic matters therein; noting the removal rate at 4 mg/l was 53% which is consistent with other results.

In summary, the aforementioned optimum result was obtained following several experiments, where initially the addition of no chemicals were explored, followed by the addition of coagulants only. Upon deriving at the best coagulant dosage, the variation of pH ranges were explored for optimum results. Then under the optimal coagulant dosage and pH range, experiments were conducted to determine the effect on removal levels at various dosages of cationic and anionic polyelectrolytes.

### Pilot Scale Study

The pilot scale study was conducted to not only confirm the results at the bench level study but to provide additional data that would aid in scaling up. It should be noted that pilot scale limit is designed and built so that the process can be better understood. It provides boundary conditions that allow scale up from the prototype to the commercial plant (Whalley 2016) [13]. A pilot plant is meant to be flexible and adaptable so that the researcher can do modifications to test configurations.

The engineering parameters critical for the run of each sample were: wastewater volume, temporal mean velocity gradient (G), tip speed and the resulting rpm for the mechanical agitators. Average volume of samples at the pilot scale was 40 L, as compared to bench scale level volume of 2 L. Two agitators were used, namely a Cole Palmer 50000-40 and a Cole Palmer 50000-60, each of propeller type, of diameter 0.0635 m, which were used both in the coagulation and flocculation tanks. In the absence of values for the “K_t_” constant normally provided by the suppliers of the mechanical agitators, use was made of the literature. As reported for Propeller type agitators with a pitch of two and 3 blades K_t_ for turbulent and laminar flows are 1 and 43.5, respectively (Tchobanglous, Burton, and Metcalf and Eddy 1991) [14]. Tip speed (TS) and Reynold’s Number were monitored to ensure all parameters remained in correct ranges (as listed in the following paragraph); however, velocity gradient was the controlling parameter. Equations for the aforementioned are as follows:

- G = √(P/(µ*V)).
- TS = 2Rὠπ.
- Power (P) = Kt/g *ν*N3*D5.
- Re =( ρND2)/µ.

The power of the stirrers are 74.5 W with an efficiency of 80% as stated by Cole Palmer. Where ideal parameter ranges are: Coagulation; G 300-1000s^− 1^,Tip Speed x > 3 ms^− 1^ and RPM < 1500. Flocculation; G 20–60 s^− 1^, Tip Speed 0.15 ms^− 1^<x < 0.6ms^− 1^, RPM x < 100 (Tchobanglous, Burton, and Metcalf and Eddy 1991) [14].

All the chemical parameters considered at the bench level study remained at the optimum dosages and strengths (PACl 250 mg/l dosage and 6 N Sodium Hydroxide for adjustment of pH at 8) except the polyelectrolytes. Polyelectrolyte dosages and strengths coupled with varying settling times were manipulated in order to attain optimum results based on limitations faced mainly by equipment type and dimensions i.e. agitator type used in flocculation tank. Subsequently, the K_t_ value and subsequent calculations for mixing were used to determine the necessary rpm to allow optimum bridging of particles, in the flocculation tank. Detention times were also varied in the first tank (Pre-sedimentation tank) to allow a proper representation of emulsified oil and grease to be treated. Time varied between thirty (30) minutes and one hundred and twenty (120) minutes with an average of one hundred and seven (107) minutes. In addition, varying strengths and dosages of the polyelectrolytes were manipulated so as to obtain the optimum results, this aids as increased strength and dosages (strength – increased functional groups /unit grams and increased dosages – number of polymer groups per unit volume of liquid) would have served by increasing the charge density for agglomeration where the mechanical aspect was insufficient for the same.

**Fig. 5:**
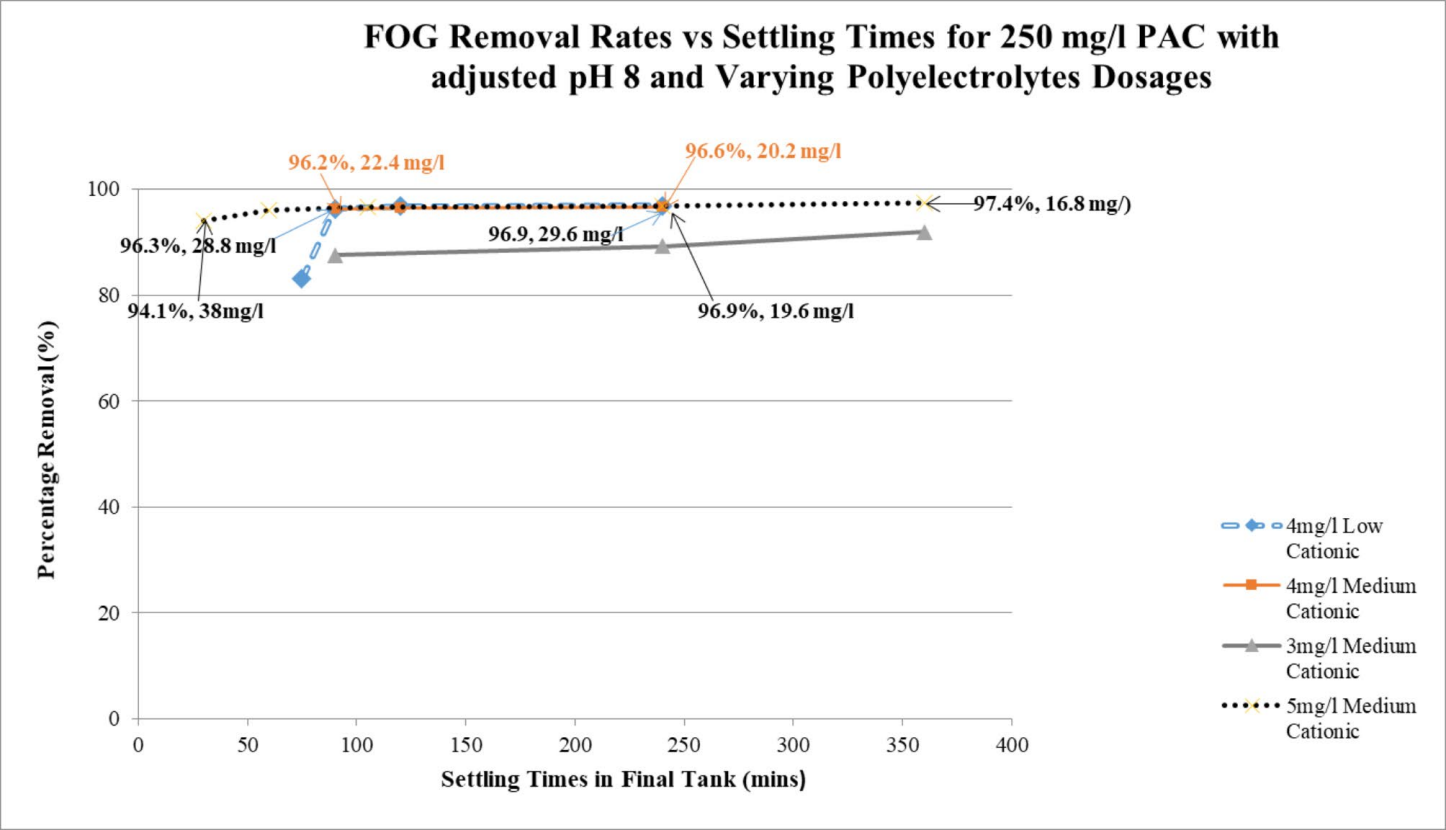
Varying Settling Times in Final Tank with Varying Polyelectrolyte Dosages and

Figure 5 illustrates the results at the pilot plant study. With all pre-determined bench level treatment parameters remaining at optimum dosages and strengths, with variation only in the polyelectrolyte dosages and strengths, and the settling times at pre sedimentation and final tanks; optimum results were attained for a dosage of 5 mg/l Medium Cationic (0.1% Stock) and a final settling time of six (6) hours respectively. Results yielded 97.4%, FOG removal and resultant residual of 16.8 mg/l. This result differs from the dosing strength for the polyelectrolyte that attained optimum results at the bench level study of 4 mg/l Low Cationic (0.1% Stock) for a removal rate of 99.9% (0.17 mg/l residual). It is concluded that the residual concentration in the bench level study is significantly below that generated at the pilot plant and may be attributed to the limitations of equipment used in the pilot plant study, as discussed above.

The secondary parameters after treatment were as follows; pH consistently remained at 7 after treatment, TSS had over 90% removals and residuals ranged between 30 mg/l and 14 mg/l. COD removal rates on the other hand were not significant ranging between 17 and 45%, as predetermined with the use of the increased strengths and dosages of cationic polyelectrolytes. The effluent had negligible amount of aluminium from operations, recorded at 0.16 mg/l, indicative of most of the aluminium in the process settled out. Formula determined concentration of aluminium in sludge to be 6999 mg/l.

### Strengths vs. % removal rates

## Conclusion

The pilot scale study confirmed findings of the bench level study, where high concentration of emulsified FOG can be efficiently removed to levels significantly below the permissible levels to allow its entry into sewer systems of Trinidad and Tobago using coagulation, flocculation and sedimentation techniques. The use of PACl at a dose of 250 mg/l, and (CP1154) low cationic polyelectrolyte at a dose of 4 mg/l, when the sample is initially adjusted to pH of 8 effectively and efficiently removed emulsified FOG to effluent levels that complied with existing standards. Subsequently, it was confirmed that the isoelectric point for aluminium hydroxide, for which optimum coagulation and flocculation to occur was 8.

Pilot scale studies allowed additional data to be obtained to explore possible boundary conditions that would aid in scaling up of the system. Here, it was revealed that a higher strength and/or dose of the cationic polyelectrolyte and increased times in primary and final tanks were required to attain the desired results as in the bench level study where limitations in the flocculation tank were faced i.e. 0.0635 m propeller agitators were used instead of a 0.2 m flat blade turbine agitators which were required for flocculation in this system. This is in alignment with theory where factors critical for agglomeration is equipment type and density charge. It is, however, believed that the optimum combination of chemicals and the respective dosages attained at the bench level study should prove very adequate should the right equipment (aforementioned) be made available.

The study also showed this pilot plant to be more economical compared to the pilot plant used in the Hong Kong study [9] i.e. Electro Coagulation and Electro Flocculation with a stimulus sequencing quadratic program. The cost comparison to run one batch in each system (present study vs. study in Hong Kong) showed coagulation/flocculation technique to be approximately USD 2653, vs. USD 8989, respectively i.e. approximately 5 times the cost, where the results both meet the permissible level for FOG based on the Trade Effluent Standards of the Authority.

An analysis of the sludge arising out of coagulation showed it to be heavily water based and high in aluminium content. As such, it can be disposed of via dewatering or digestion and any remnants can be disposed of via the Caroni Water Treatment Plant’s (The largest water treatment plant in Trinidad and Tobago that disposes of bioproducts from its processes such as alum) Sludge Ponds which has internal methods of disposing of this type of sludge waste. Research on sludge with this concentration of aluminium also reveals that it can be used for cement mortar and concrete making (Chakraborty 2005). The literature review has shown the other two layers of FOG i.e. floatables and settleables can be disposed of via acid digestion or based on its property it can be incinerated using controlled mechanisms.

In the present study, it has been experimentally substantiated that physico-chemical (coagulation-flocculation-sedimentation) method of treatment of effluents from FSEs is an environmentally friendly and viable option for haulers to dispose of grease laden wastewater in a safe manner. It eliminates the problems of illegal dumping and the associated hazards. Other quality parameters such as aluminium content, TSS and pH also satisfy to be conforming to the stipulated standards to allow the treated wastewater to enter into the sewer systems. COD removal was low, due to the polymeric properties therein of the polyelectrolyte.

It should be noted that Biochemical Oxygen Demand (BOD_5_) was not monitored in the treatment phase due to time constraints. In addition, in Trinidad and Tobago, reduction of BOD_5_ is one of the primary objectives met at the wastewater treatment plants, whilst FOG isn’t. Based on the aforementioned discussion, it can be concluded that the engineering solution for disposal of FOG from FSEs in Trinidad has been made possible and all consequential issues could also be addressed through various proposed engineering options.

## Data Availability

The datasets used and/or analysed during the current study are available from the corresponding author upon reasonable request.

## List of abbreviations

BOD_5_: Biochemical Oxygen Demand
COD: Chemical Oxygen Demand
EMA: Environmental Management Authority
FOG: Fats Oils and Grease
FTIR: Fourier Transforms Infrared Spectroscopy
FSEs: Food Services Establishments
GI: Grease Interceptors
HLD: High Level Disinfection
IX: Ion Exchange
MF: Micro Filtration
PACl: Poly-aluminium Chloride
pH: Power of Hydrogen
RO: Reverse Osmosis
RPM: Revolutions per Minute
SWMCOL: Solid Waste Management Company Limited
TES: Trade Effluent Standards
TSS: Total Suspended Solids
UV-A: Ultraviolet Disinfection with Advanced Oxidation (UV-A).
WASA: Water and Sewerage Authority
WWTP: Wastewater Treatment Plant
