# Correction: Treatment of effluents from Food services establishment (FSEs) by physico-chemical processes: a case study for Trinidad & Tobago

**DOI:** 10.1186/s13036-024-00474-9

**Published:** 2025-03-10

**Authors:** Cudjoe Shamika, Banerjee Goutam, Cooper Vincent

**Affiliations:** 1 Department of Civil and Environmental Engineering, The University of The West Indies, St. Augustine, Trinidad & Tobago; 2 Department of Civil Engineering, Indian Institute of Technology (IIT), Varanasi, 221005 India

**Correction: Journal of Biological Engineering (2024) 18:2**

10.1186/s13036-023-00344-w

Minor corrections were deemed necessary to rectify typographical errors that had occurred in the original article [1].

The changes do not affect the scientific content of the article, which has now been corrected.
